# Gut Microbiota Dysbiosis in Human Hypertension: A Systematic Review of Observational Studies

**DOI:** 10.3389/fcvm.2021.650227

**Published:** 2021-05-14

**Authors:** Yang Guo, Xiaosu Li, Zhijian Wang, Bo Yu

**Affiliations:** ^1^Department of Dermatology, Skin Research Institute of Peking University Shenzhen Hospital, Peking University Shenzhen Hospital, Shenzhen Peking University-The Hong Kong University of Science and Technology Medical Center, Shenzhen, China; ^2^Department of Cardiology, Peking University Shenzhen Hospital, Shenzhen, China

**Keywords:** hypertension, gut microbiota, metabolism, short chain fatty acids, humans

## Abstract

**Introduction:** Hypertension is one of the major risk factors to human health and human studies on association between gut microbiota and hypertension or blood pressure have received increased attention. In the present study, we aim to evaluate gut microbiota dysbiosis in human hypertension using a method of systematic review.

**Methods:** PubMed, EMBASE, and Web of Science databases were searched until March 2021 to identify eligible articles. Additional articles were also identified by searching specific authors in this field. Inclusion criteria were observational studies based on stool samples with hypertension group and control group. Newcastle-Ottawa quality assessment scale (NOS) was used to assess the quality of the included studies. PROSPERO registration number: CRD42020212219.

**Results:** A total of 17 studies enrolling 9,085 participants were included. Fifteen of the enrolled studies showed good quality and two studies showed fair quality based on NOS. We found alpha diversity in hypertension decreased significantly and microbial structure can be separated compared with control groups. Gut microbiota of hypertension showed depletion of short chain fatty acids (SCFAs) producers and over-growth of some *Proteobacteria* and *Bacteroidetes* members. Up-regulation of lipopolysaccharide biosynthesis, phosphotransferase system, ABC transporters, etc. and down-regulation of some amino acid metabolism, etc. in hypertension were reported. Fecal SCFAs levels increased and plasma SCFAs levels decreased in hypertension. Stronger microbial interactions in hypertension were seen.

**Conclusion:** In conclusion, gut microbiota dysbiosis was observed in hypertension, including decreased diversity, altered microbial structure, compositional change of taxa, alterations of microbial function, nutritional and immunological factors, and microbial interactions. Poor absorption and high excretion of SCFAs may play an important role in the pathogenesis of hypertension. These findings may provide insights into etiology study and new microbial-based therapies of hypertension.

**Systematic Review Registration:** PROSPERO database, identifier CRD42020212219.

## Introduction

Hypertension is a major risk factor of cardiovascular, cerebrovascular, and kidney disorders, leading to heavy disease burden. Globally, ~1.13 billion cases in 2015 were reported and the increase was seen largely in low-income and middle-income countries (1). Etiology studies have shown that hypertension is a complex and multifactorial disorder influenced by various factors, including genetic factors (2, 3) and lifestyle factors (4), such as diet, obesity, physical inactivity, etc. However, based on a large-scale genome-wide association study, blood pressure–associated SNPs can only explain 3.46 and 3.36% of the variance in systolic blood pressure (SBP) and diastolic blood pressure (DBP), respectively (2), indicating that other potential risk factors remain to be explored. In terms of treatment, despite multiple options, treatment resistant hypertension is identified in about 20% of hypertensive cases, with few treatment options (5). Therefore, novel therapeutic approaches for the treatment of hypertension are needed.

There are increasing evidences that gut microbiota dysbiosis is associated with many disorders, one of which is hypertension. Gut microbiota is key to human health, including immune system, metabolism and nutrition benefit, and maintaining healthy of gut microbiota is essential to human health (6, 7). Studies suggested that gut microbiota and its metabolites play a critical role in blood pressure regulation. Short-chain fatty acids (SCFAs), important metabolites of gut microbiota, could affect immune system and epithelial functions in the modulation of blood pressure (8). Meta-analyses showed that higher consumption of fiber, which can be fermented by gut bacteria as a source of SCFAs, was associated with decreased blood pressure (9, 10). Human studies on association between gut microbiota and hypertension or blood pressure have received increased attention; it is therefore warranted to summarize the available literatures for understanding the role of gut microbiota in hypertension pathogenesis and offering novel insights for preventive and treatment strategies.

In the present study, we systematically reviewed the current available human studies assessing gut microbiota alterations in hypertension and summarized major findings in this field, providing clues for future studies on etiology and targeted therapy in aspect of gut microbiota.

## Methods

This systematic review was designed and reported according to the recommendations of the Preferred Reporting Items for Systematic Reviews and Meta-Analyses (PRISMA statement) (11). The study was registered with the PROSPERO database (Registration number: CRD42020212219).

### Literature Search and Study Selection

A systematic search was performed based on PubMed, EMBASE, and Web of Science on September 19, 2020 and updated on March 26, 2021. The full search strategies can be found in Supplementary Table 1. Additionally, other data sources were also considered including contact with experts and review of references cited in the included papers.

The eligibility criteria for study inclusion were established according to the PICOS strategy:

1. Participants/population: people with and without hypertension and with gut microbiota measured (fecal samples);
2. Interventions/exposures: hypertension or blood pressure;
3. Comparators/controls: hypertension group vs. control group;
4. Outcomes: (1) primary outcome: changes of microbiota diversity and differential microbes between hypertension and controls; (2) secondary outcome: major findings of microbiota functions, nutritional and immunological factors, and microbial interactions;
5. Study design: observational studies with hypertension group and control group.

Additionally, the search strategy did not include language restriction; the articles which were not published in English or Chinese and did not provide English or Chinese abstracts were excluded. Conference abstracts were excluded as limited information was reported. Two researchers (Y. G. and X. L.) independently did the literature search and study selection. Discrepancies were resolved through group discussions.

### Data Extraction

Using pre-designed standardized data abstraction forms, two researchers (Y. G. and X. L.) independently extracted characteristics of include studies, with any disagreements resolved by consensus. The extracted information included: authors, publication year, journal, region, study design, method of characterization of the microbiota, major findings, etc.

### Assessment of Risk of Bias in Included Studies

Newcastle-Ottawa quality assessment scale (NOS) (12) was used to assess the quality of the included studies. A star system has been developed in which a study is judged on three broad perspectives: (1) Selection, (2) Comparability, and (3) Exposure/ Outcome. A study can be awarded a maximum of one star for each numbered item within the Selection and Exposure categories; a maximum of two stars can be given for Comparability. Totally, a maximum of nine stars can be given for a study and more stars represent higher quality. Studies were categorized as good quality if the score was ≥7, fair quality if the score was 4~6, and poor quality if the score was <4 (13).

### Outcome Assessment

The pre-specified primary outcomes were changes of microbiota diversity and differential microbes between hypertension and controls. Alpha diversity is a quantitative measure of community diversity and beta diversity is a measure of similarity between samples (14). Alterations of diversity are associated with perturbations or imbalance in the community composition (generally referred to as dysbiosis). Detection of differential microbes between groups is an exploration of key taxa, which could be potential biomarkers, and this may offer clues for downstream interventional trials (15).

The secondary outcomes were major findings of microbiota functions, nutritional and immunological factors, and microbial interactions. Findings of microbiota functions predicted using Phylogenetic Investigation of Communities by Reconstruction of Unobserved States (PICRUSt) were summarized and may provide clues for further validation study (16). The nutritional and immunological findings, which are intimately related to the gut microbial profile, include SCFAs, cytokines, inflammatory compounds/anti- inflammatory compounds, immune cells, etc. (7). Furthermore, the microbial interaction analysis may illustrate how microbes interact with each other and the dynamic changes in the pathogenesis of hypertension (17).

## Results

### Characteristics of Included Studies

**Figure 1:**
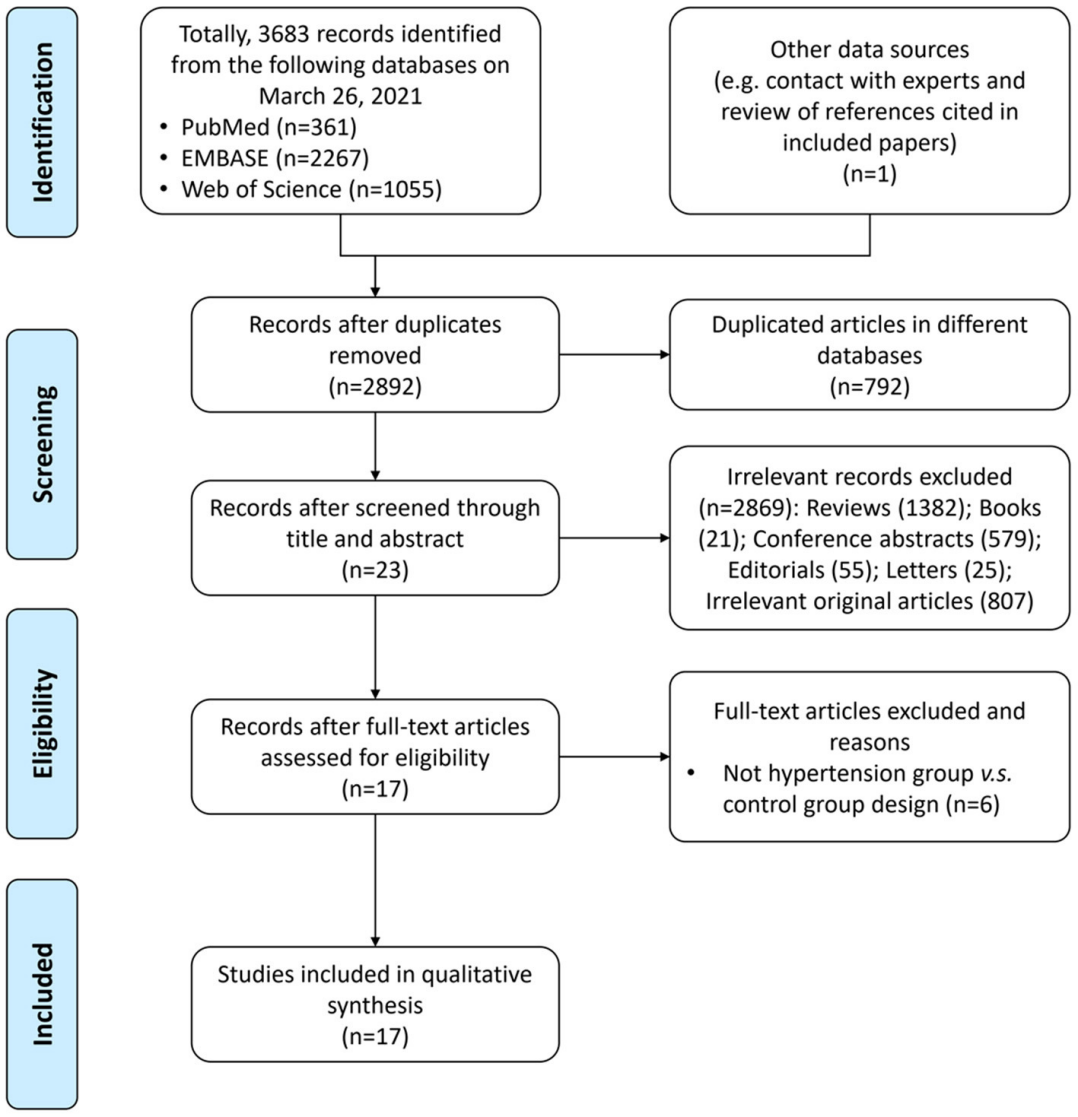
PRISMA flow diagram for study selection.

A total of 3,683 records were identified from the three databases, including PubMed, EMBASE, and Web of Science, and one record was identified by searching publications of specific experts; 792 of them were repeatedly included from more than one search database and were excluded. Totally, 17 articles (18–34) with 9,085 participants were included in the final analysis. The study selection process is shown in the PRISMA flow diagram (Figure 1).

The characteristics of included studies were summarized in Table 1. Most of them were published in year 2019 (6/17 studies) (23–28) and year 2020 (6/17 studies) (29–34); in terms of geographical region, most of these researches were conducted in China (9/17 studies) (18–21, 23, 25, 27, 28, 30) and the US (3/15 studies) (22, 27, 31), which are two countries with heavy disease burden of hypertension. For study design, most studies enrolled a group of fecal samples from hypertension cases and a group of fecal samples from healthy controls with normal blood pressure; the major exploration was based on comparison between the two groups; three studies (18, 20, 24) also enrolled subjects of pre-hypertension or in borderline status. Additionally, one study (30) focused on preeclampsia and another one (31) focused on pulmonary arterial hypertension (PAH). As for evaluation method of microbiota, most studies (23–27, 29, 30, 33, 34) (9/17 studies) used 16S ribosomal RNA gene sequencing and seven studies (18, 19, 21, 22, 28, 31, 32) used metagenomic sequencing. Moreover, one study (20) utilized viral sequencing to profile the gut virome.

**Table 1:** Characteristics of the included studies.

**Study**	**Country**	**Cases**	**Controls**	**Total sample size**	**Evaluation of microbiota**
**Hypertension**					
Silveira-Nunes et al. (33)	Brazil	Reported and treated hypertension (SBP >140 mmHg and DBP >90 mmHg) for more than 10 years (*n =* 48, mean age: 65.3 ± 15.5 y, male/female: 14:34)	Those with no report of hypertension (*n =* 32, mean age: 63.3 ± 15.0 y, male/female: 7:25)	80	16S rRNA gene sequencing
Palmu et al. (32)	Finland	SBP ≥140 mm Hg, DBP ≥90 mm Hg, or use of antihypertensive medication (*n =* 3,291, mean age for all subjects: 49.2 ± 12.88 y, male/female for all subjects: 3,819:3,134)	Normotensive individuals from the same cohort (*n =* 3,662, mean age for all subjects: 49.2 ± 12.88 y, male/female for all subjects: 3,819:3,134)	6,953	Metagenomic sequencing
Calderon-Perez et al. (29)	Spain	SBP between 140 and 159 mmHg and were not using antihypertensive treatment (*n =* 29, mean age: 53.7 ± 9.6 y, male/female: 19:10)	SBP <120 mmHg (*n =* 32, mean age: 41.1 ± 9.1 y, male/female: 16:16)	61	16S rRNA gene sequencing
Takagi et al. (34)	Japan	SBP ≥140 mm Hg, DBP ≥90 mm Hg, or current use of antihypertensive drugs (*n =* 97, median age: 69 (37–87) y, male/female: 49:48)	Healthy controls without gastrointestinal inflammatory diseases or functional gastrointestinal disorders, without use of antibiotics, corticosteroids, immunosuppressants, or acid-suppressing agents within the past 3 months, and without as a history of underlying malignant disease (*n =* 54, median age: 65.5 (16–88) y, male/female: 21:33)	151	16S rRNA gene sequencing
Sun et al. (27)	US	Current use of antihypertensive medication, an SBP ≥140 mm Hg, or a diastolic BP ≥90 mm Hg (*n =* 186, mean age for all subjects: 55.3 ± 3.4 y, male/female for all subjects: 244:285)	Healthy controls (*n =* 343, mean age for all subjects: 55.3 ± 3.4 y, male/female for all subjects: 244:285)	529	16S rRNA gene sequencing
Mushtaq et al. (26)	China	Grade 3 hypertension (according to the World Health Organization BP classification) (*n =* 50, mean age: 62.5 ± 10.4 y, male/female: 28/22)	Healthy controls with no history of hypertension, or any other cardiovascular or chronic metabolic disease (*n =* 30, mean age: 60.5 ± 11 y, male/female: 16/14)	80	16S rRNA gene sequencing and quantitative PCR
Dan et al. (23)	China	SBP ≥140 mmHg or DBP ≥90 mmHg (*n =* 62, mean age: 69.322 ± 10.613 y, male/female: 0:62)	90 mmHg ≤ SBP ≤ 140 mmHg and 60 mmHg ≤ DBP ≤ 90 mmHg (*n =* 67, mean age: 69.492 ± 9.630 y, male/female: 0:67)	129	16S rRNA gene sequencing
Li et al. (25)	China	SBP ≥140 mmHg, DBP ≥90 mmHg, or by self-reported use of antihypertensive medications in the last 2 weeks irrespective of BP valuesNaive hypertension (*n =* 63, mean age: 58.4 ± 10.2 y, male/female: 35/28)Anti-hypertensive (*n =* 104, mean age: 59.8 ± 9.3 y, male/female: 50:54)	Healthy controls (*n =* 42, mean age: 59.3 ± 9.2 y, male/female: 17:25)	209	16S rRNA gene sequencing
Zuo et al. (28)	China	SBP >140 mm Hg and DBP >90 mm Hg (*n =* 34, median age: 54.5 (49.3–57.8) y, male/female: 31/3)	Healthy controls (*n =* 15, median age: 58.0 (52.0–60.0) y, male/female: 11:4)	49	Metagenomic sequencing
Kim et al. (22)	US	SBP ≥140 mmHg (*n =* 22, not report characteristics of final included subjects)	SBP ≤ 130 mmHg irrespective of subject's antihypertensive drug regimen (*n =* 18, not report characteristics of final included subjects)	40	Metagenomic sequencing
Jin et al. (21)	China	SBP ≥140 mmHg or DBP ≥90 mmHg (*n =* 73, mean age: 53.7 ± 5.7 y, male/female: 66:7)	SBP <120 mmHg and DBP <80 mmHg (*n =* 68, mean age: 52.5 ± 5.7 y, male/female: 59:9)	141	Metagenomic sequencing
Yan et al. (19)	China	Primary hypertension: current blood pressure ≥140/90 mm Hg (*n =* 60, mean age: 57.0 ± 9.6 y, male/female: 35/25)	Gender-, age-, and body weight-matched healthy controls (current blood pressure ≤ 120/80 mm Hg) (*n =* 60, mean age: 56.0 ± 8.6 y, male/female: 32:28)	120	Shotgun metagenomics
**Hypertension and borderline hypertension/Pre-hypertension**					
Huart et al. (24)	Belgium	**Hypertension:** mean 24-h SBP levels ≥130 mm Hg or DBP ≥80 mm Hg or in case of use of antihypertensive medications whatever the BP levels (*n =* 38, mean age: 52.5 ± 8.2 y, male/female: 38:0) **Borderline hypertension:** mean 24-h BP levels <130/80 mm Hg with either isolated daytime hypertension (SBP ≥135 mm Hg and DBP ≥85 mm Hg) or nocturnal hypertension (SBP ≥120 mm Hg or DBP ≥70 mm Hg) (*n =* 7, mean age: 50.3 ± 13.3 y, male/female: 7:0)	Untreated individuals with mean 24-h BP levels <130/80 mm Hg (*n =* 9, mean age: 46.2 ± 11.4 y, male/female: 9:0)	54	16S rRNA gene sequencing
Han et al. (20)	China	**Hypertension patients:** SBP ≥140 mmHg, or DBP ≥90 mmHg without antihypertensive treatments (*n =* 99, not report characteristics of final included subjects) **Pre-hypertension persons:** 125 mmHg < SBP ≤ 139 mmHg or 80 mmHg < DBP ≤ 89 mmHg without antihypertensive treatments (*n =* 56, not report characteristics of final included subjects)	SBP ≤ 125 mmHg and DBP ≤ 80 mmHg without antihypertensive treatments (*n =* 44, not report characteristics of final included subjects)	199	Viral sequencing
Li et al. (18)	China	**Hypertension patients:** 140 mmHg ≤ SBP, or 90 mmHg ≤ DBP (*n =* 99, mean age: 53.6 ± 5.5 y, male/female: 93:6) **Pre-hypertension persons:** 125 mmHg < SBP ≤ 139 mmHg, or 80 mmHg < DBP ≤ 89 mmHg (*n =* 56, mean age: 51.8 ± 6.6 y, male/female: 52:4)	SBP ≤ 125 mmHg, or DBP ≤ 80 mmHg (*n =* 41, mean age: 53.7 ± 5.9 y, male/female: 32:9)	196	Metagenomic sequencing
**Preeclampsia**					
Chang et al. (30)	China	An elevated systolic BP of ≥160 mm Hg or a diastolic BP of ≥110 mm Hg, proteinuria of ≥3 g/24 h (*n =* 27, mean age: 31.7 ± 4.9 y, male/female: 0:27)	Healthy pregnant control (*n =* 36, mean age: 30.4 ± 4.1 y, male/female: 0:36)	63	16S rRNA gene sequencing
**Pulmonary arterial hypertension**					
Kim et al. (31)	US	Type 1 PAH patients (mean pulmonary arterial pressure 57.4 ± 16.7 mmHg) (*n =* 19, mean age: 39.5 ±12.7 y, male/female: 3/16; final include: *n =* 18)	Age- and sex-matched healthy controls (*n =* 16, mean age: 37 ± 11 y, male/female: 3/13; final included: *n =* 13)	31	Metagenomic sequencing

*DBP, diastolic blood pressure; NOS, Newcastle–Ottawa quality assessment scale; PAH, pulmonary arterial hypertension; SBP, systolic blood pressure*.

### Risk of Bias in Included Studies

**Figure 2:**
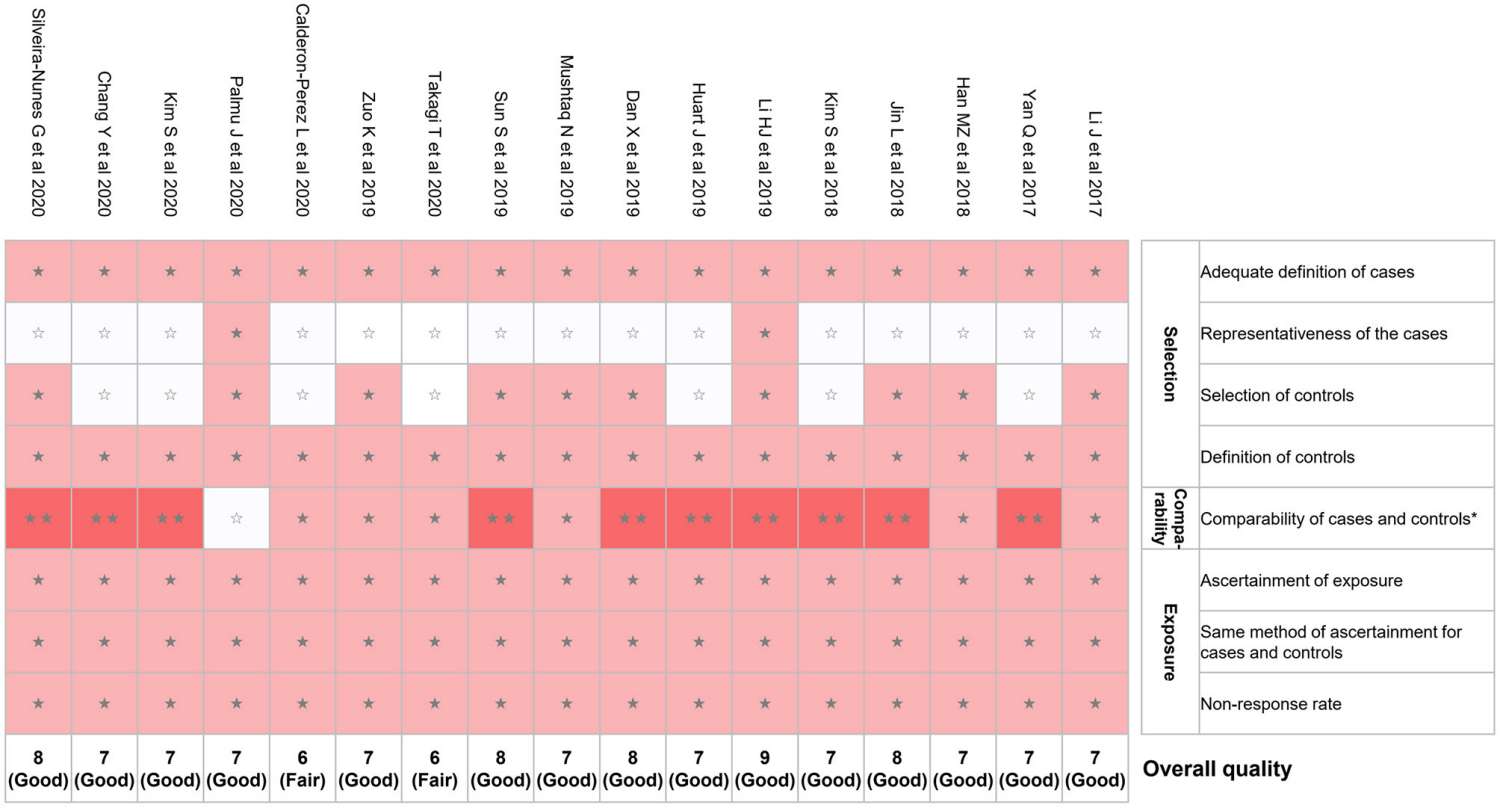
Risk of bias assessment using NOS. *A maximum of 2 stars can be given in this category: one for age and the other one for other controlled factors. NOS, Newcastle–Ottawa quality assessment scale. ⋆⋆means 2 points were given; ⋆means 1 point was given; means 0 point was given.

We evaluated the risk of bias for the 17 studies using NOS (Figure 2). The mean NOS score of included studies in the present systematic review was 7.2 ± 0.7; 15 out of 17 studies showed good quality. However, potential selection bias was observed due to the lack of representativeness of the cases in 15 of 17 studies (Figure 2).

### Change of Gut Microbiota Diversity in Hypertension

Thirteen articles reported the change of alpha diversity in hypertension (Table 2). Some studies showed alpha diversity indexes were decreased in fecal samples of hypertension (18, 19, 28, 33), pre-hypertension (18), preeclampsia (30), and PAH (31). Another detailed multivariable-adjusted regression analysis of the association between alpha diversity indexes and blood pressure indicated that blood pressure was inversely associated with measures of alpha-diversity (27). For Shannon index, in multivariable-adjusted models between gut microbial alpha diversity and hypertension, odds ratios (ORs) were ranging from 0.82 to 0.90 in different models; for Richness index, ORs were ranging from 0.70 to 0.79 in different models (27). However, some studies suggested that no significant differences in alpha diversity indexes were seen between hypertension and controls (23, 29), including the virome study (20); similarly, another multivariable-adjusted regression analysis (32) reported that alpha diversity was not related to any BP variable. Additionally, one study (26) reported that Chao 1 index and abundance-based coverage estimator (ACE) index were higher in hypertension group than controls group. Notably, different indexes representing alpha diversity may show different change trends in one single study (26, 28, 33). For instance, in one study (33), Shannon index was decreased in hypertension group; but no significant differences were observed in phylogenetic diversity index and OTUs count between hypertension group and control group.

**Table 2:** Major findings of the included studies on diversity.

**Study**	**Change of alpha diversity in hypertension**	**Change of beta diversity in hypertension**
**Hypertension**		
Silveira-Nunes et al. (33)	Shannon index was decreased in hypertension group; phylogenetic diversity and OTUs count were similar in hypertension group and control group	Significant differences between hypertension group and control group were detected
Palmu et al. (32)	Alpha diversity was not related to any BP variable in the multivariable-adjusted models	In multivariable-adjusted models, beta diversity was only associated with DBP
Calderon-Perez et al. (29)	No significant differences of Shannon index and Chao 1 were seen between different groups	No significant differences were detected
Takagi et al. (34)	No significant differences of Shannon index and the observed species were seen between different groups	No significant differences were detected
Sun et al. (27)	Hypertension and SBP were inversely associated with measures of alpha-diversity, including Richness (ORs: 0.70~0.79 in different models) and the Shannon Diversity Index (ORs: 0.82~0.90 in different models)	Beta diversity was significantly associated with both hypertension and SBP in all multivariable-adjusted models
Mushtaq et al. (26)	Observed species, OTUs, Shannon, Simpson, and Good's coverage were similar in hypertension group and control group; chao1 and ACE were higher in hypertension group than controls group	Significant differences between hypertension group and control group were detected
Dan et al. (23)	No significant differences were seen between hypertension group and control group	Significant differences between hypertension group and control group were detected
Zuo et al. (28)	Shannon index and Pielou evenness were decreased in hypertension group; Chao richness was similar in hypertension group and control group	-
Kim et al. (22)	-	Samples were separate significantly based on bacterial taxonomy
Yan et al. (19)	Shannon index was decreased in hypertension group	Overlaps were seen only in part with taxonomic composition based on Bray-Curtis distances
**Hypertension and borderline hypertension/Pre-hypertension**		
Han et al. (20)	No significant differences were seen between different groups	Samples were stratified into two viral-types
Li et al. (18)	Shannon index was decreased in hypertension group and pre-hypertension group	Samples were clustered into two enterotypes by PCA of Jensen-Shannon divergence
**Preeclampsia**		
Chang et al. (30)	Shannon index and Sobs index were decreased in hypertension group	Significant differences between hypertension group and control group were detected
**Pulmonary arterial hypertension**		
Kim et al. (31)	Shannon index, Simpson's index and Evenness were decreased in PAH group	Significant differential taxonomic profiles between PAH and control group were observed

*BP, blood pressure; DBP, diastolic blood pressure; OR, odds ratio; OTUs, operational taxonomic units; PAH, pulmonary arterial hypertension; SBP, systolic blood pressure*.

As for beta diversity (microbial community structure), 13 studies reported related findings (Table 2). Most studies (11/13 studies) showed that significant differences between hypertension group and control group were detected (18–20, 22, 23, 26, 27, 30–33). Two studies reported that no significant differences were seen (29, 34).

### Key Taxa Associated With Hypertension

The major taxa with disparate representation in hypertension were summarized in Table 3. Overall, these studies reported heterogeneous results; therefore, we focus on key taxa which were reported with consistent change across at least two different studies. First, gut microbiota of hypertension showed over-growth of *Megasphaera* (23, 25, 26), *Prevotella* (18, 26), *Klebsiella* (18, 19), *Parabacteroides* (21, 23), *Alistipes* (23, 25), *Enterobacter* (30, 32), *Escherichia* (26, 30), *Shigella* (26, 30), etc. Whereas, *Faecalibacterium* (18, 26, 28, 33), *Roseburia* (18, 19, 28, 33), *Ruminococcus* (23, 27), *Coprococcus* (18, 31), *Butyrivibrio* (18, 28, 31), *Bifidobacterium* (18, 30), *Sporobacter* (23, 27), *Akkermansia* (27, 28, 31), *Bacteroides* (26, 31), *Eubacterium rectale* (22, 30), *Faecalibacterium prausnitzii* (19, 29), *Clostridium* (23, 28), *Marvinbryantia* (23, 28), *Subdoligranulum* (28, 30), *Anaerotruncus* (23, 28), *Intestinimonas* (23, 28), etc. were depleted in hypertension. In short, SCFA producers, including *Faecalibacterium, Roseburia, Ruminococcus, Bifidobacterium, Akkermansia*, and *Bacteroides*, etc. were depleted in hypertension; on the contrary, some *Proteobacteria* and *Bacteroidetes* members were enriched in hypertension, such as *Klebsiella, Prevotella*, and *Enterobacter*.

**Table 3:** Major taxa with disparate representation in hypertension.

**Kingdom**	**Phylum**	**Class**	**Order**	**Family**	**Genus**	**Species**
Bacteria	Firmicutes	Bacilli	Lactobacillales	*Lactobacillaceae*	*Lactobacillus*↑ (33)↓ (23)	*Lactobacillus rhamnosus*↑ (32)*Lactobacillus farciminis*↓ (32)
				*Streptococcaceae*	*Streptococcus*↑ (19) ↓ (30)	
					*Lactococcus*↑ (25)	
				*Enterococcaceae*	*Enterococcus*↓ (23)	
			Bacillales	*Paenibacillaceae*	*Paenibacillus* ↓ (28)	
		Clostridia	Clostridiales	*Clostridiaceae*	*Clostridium*↑ (24) ↓ (23, 28)	
					*Hungatella*↓ (21)	
					*Butyricicoccus*↓ (28)	
				*Lachnospiraceae*↓ (31)	*Roseburia*↓ (18, 19, 28, 33)	*Roseburia hominis*↓ (29)
					*Blautia*^b^↓ (30)	
					*unclassified Lachnospiraceae*↑ (29)	*Eubacterium rectale*↓ (22, 30)
					*Anaerobutyricum*	*Eubacterium hallii*^b^↓ (30)
					*Coprococcus*↓ (18, 31)	
					*Butyrivibrio*↓ (18, 28, 31)	
					*Eisenbergiella*↑ (23)	
					*Cellulosilyticum*↓ (23)	
					*Marvinbryantia*↓ (23, 28)	
					*Sporobacterium*↓ (23)	
					*Dorea*	*Dorea unclassified*↑ (22)
					*Oribacterium*↓ (28)	
					*Robinsoniella*↓ (28)	
				*Ruminococcaceae*	*Faecalibacterium*↓ (18, 26, 28, 33)	*Faecalibacterium prausnitzii*↓ (19, 29)
					*Ruminococcus*↓ (23, 27)	*Ruminococcus callidus*↓ (22)
					*Subdoligranulum*↑ (25)↓ (28, 30)	
					*Gemmiger*↓ (23)	
					*Flavonifractor*↓ (23)	
					*Anaerotruncus*↓ (23, 28)	
					*Sporobacter*↓ (23, 27)	
					*Ruminococcaceae_NK4A214*↓ (29)	
					*Ruminococcaceae_UCG-010*↓ (29)	
					*Pseudoflavonifractor* ↓ (28)	
				*Eubacteriaceae*	*Eubacterium*^a^↓ (31)	
				*Christensenellaceae*	*Christensenellaceae_R-7*↓ (29)	
					*Christensenella*↑ (23)	
				*Clostridiales Family XIII. Incertae Sedis*	*Anaerovorax*↓ (27)	
					*Acidaminobacter*↓ (23)	
					*Guggenheimella*↓ (23)	
				*Oscillospiraceae*	*Oscillibacter*↓ (18, 28)	
				*unclassified Clostridiales*	*Proteiniborus*↑ (23)	
					*Intestinimonas*↓ (23, 28)	
				*Peptostreptococcaceae*	*Terrisporobacter*↑ (23)	
					*Romboutsia*↓ (23)	
				*Hungateiclostridiaceae*	*Ruminiclostridium* ↓ (28)	
		Negativicutes	Veillonellales	*Veillonellaceae*	*Veillonella*↑ (27) ↓ (23)	*Veillonella unclassified*↓ (22)
					*Megasphaera*↑ (23, 25, 26)	
					*Dialister*↑ (23)	
			Selenomonadales	*Selenomonadaceae*	*Megamonas*↑ (25)	
					*Mitsuokella*↑ (23)	
			Acidaminococcales	*Acidaminococcaceae*	*Phascolarctobacterium*↓ (23)	
		Erysipelotrichia	Erysipelotrichales	*Erysipelotrichaceae*	*Faecalitalea*↑ (23)	
					*Bulleidia*↓ (23)	
					*Holdemania* ↓ (28)	
	Bacteroidetes	Bacteroidia	Bacteroidales	*Rikenellaceae*	*Alistipes*↑ (23, 25)↓ (30)	*Alistipes finegoldii*↑ (22)*Alistipes indistinctus*↑ (22)
					*Acetobacteroides*↑ (23)	
				*Bacteroidaceae*	*Bacteroides*↑(23)↓ (26, 31)	*Bacteroides coprocola*↑ (29)*Bacteroides plebeius*↑ (29)*Bacteroides thetaiotaomicron*↓ (22)*Bacteroides salyersiae*↓ (22)
				*Barnesiellaceae*	*Barnesiella*↑ (23)	
					*Coprobacter*↓ (23)	
				*Prevotellaceae*	*Prevotella*↑ (18, 26)↓ (23)	
					*Paraprevotella*↓ (23)	*Paraprevotella xylaniphila*↓ (22)*Paraprevotella clara*↓ (22)
				*Tannerellaceae*	*Parabacteroides*↑ (21, 23)	*Parabacteroides merdae*↑ (19)
				*Porphyromonadaceae*	*Porphyromonas*↑ (18)	
					*Macellibacteroides*↓(23)	
				*Odoribacteraceae*	*Butyricimonas*↑ (23)	
	Proteobacteria	Gammaproteo bacteria	Enterobacteriales	*Enterbacteriaceae*	*Enterobacter*↑ (30, 32)↓ (21)	
					*Escherichia*↑ (26, 30)	
					*Shigella*↑ (26, 30)	
					*Kluyvera*↑ (32)	
					*Klebsiella*↑ (18, 19)↓ (22)	
				*Morganellaceae*	*Cosenzaea*↑ (23)	
		Betaproteobacteria	Burkholderiales	*Sutterellaceae*	*Sutterella*↓ (23)	
					*Parasutterella*↑ (26) ↓ (23)	
			Neisseriales	*Chromobacteriaceae*	*Microvirgula*↑ (23)	
		Deltaproteobacteria	Desulfovibrionales	*Desulfovibrionaceae*	*Desulfovibrio*↑ (23)	
		Alphaproteobacteria	Rhodospirillales	*Acetobacteraceae*	*Acidiphilium* ↓*(28)*	
	Verrucomicrobia	Verrucomicrobiae	Verrucomicrobiales	*Akkermansiaceae*	*Akkermansia*↑ (33)↓ (27, 28, 31)	
	Actinobacteria	Actinobacteria	Actinomycetales	*Actinomycetaceae*	*Actinomyces*↑ (18)	
			Bifidobacteriales	*Bifidobacteriaceae*	*Bifidobacterium*↑ (31)↓ (18, 30)	
		Coriobacteriia	Coriobacteriales	*Coriobacteriaceae*	*Collinsella*↑ (34)↓ (30)	*Collinsella aerofaciens*^a^↑ (31)
				*Atopobiaceae*	*Olsenella*↓ (23)	
			Eggerthellales	*Eggerthellaceae*	*Asaccharobacter*↓ (27)	
					*Adlercreutzia*↓ (23)	
					*Enterorhabdus*↓ (23)	
Viruses	-	*-*	*-*	*Baculoviridae*	*Betabaculovirus*	*Cnaphalocrocis medinalis granulovirus*↑ (20)
	Uroviricota	Caudoviricetes	Caudovirales	*Siphoviridae*	*Moineauvirus*	*Streptococcus virus phiAbc2*↓ (20)
					*Kostyavirus*	*Mycobacterium phage Toto*↓ (20)
					*Ceduovirus*	*Lactococcal phages*↓ (31)
					*unclassified Siphoviridae*	*Enterococcal phages*↑ (31)
				*Podoviridae*	*Lederbergvirus*	*Salmonella phage vB SemP Emek*↓ (20)

a *Altered specifically in PAH*;

b *Altered specifically in preeclampsia*.

Regarding the two studies, which were focusing on PAH (31) and preeclampsia (30), we summarized specific bacteria reported concerning PAH and preeclampsia in genus and species levels (Table 3). We found that *Collinsella aerofaciens* (species) enriched and *Eubacterium* (genus) depleted specifically in PAH; for preeclampsia, it was shown that *Blautia* (genus) and *Eubacterium hallii* (species) depleted specifically in preeclampsia.

### Alterations of Gut Microbial Function in Hypertension

Nine studies performed function analyses of gut microbiota using PICRUSt (Table 4). Similarly, these studies yielded different results for microbial function modification in hypertension and we summarized the common ones. Up-regulation of lipopolysaccharide (LPS) biosynthesis (18, 19, 22, 30), phosphotransferase system (PTS) (18, 22), ABC transporters (21, 22), and down-regulation of some amino acid metabolism (18, 19) in hypertension were reported.

**Table 4:** Major findings of microbiota functions, nutritional and immunological factors, and microbial interactions.

**Study**	**Gut microbial functional alterations**	**Nutritional and immunological factors alterations**	**Gut microbial interactions alterations**
**Hypertension**			
Silveira-Nunes et al. (33)	-	Plasma levels of TNF, IL-6, and TNF/IFN-γ ratio were increased in hypertension	-
Palmu et al. (32)	Most prominent pathways were related to lipid metabolism, gluconeogenesis, and xenobiotic metabolism, etc.	-	-
Calderon-Perez et al. (29)	**Up-regulated in hypertension:** Electron transport energy metabolism, anaerobic energy metabolism, DNA transformation, DNA replication, recombination and repair **Down-regulated in hypertension:** Signal Transduction_Two Component Systems	Fecal SCFAs levels were higher and plasma SCFAs levels were lower in hypertension	-
Takagi et al. (34)	**Up-regulated in hypertension:** Metabolic enzyme families and environmental information processing membrane transport **Down-regulated in hypertension:** Genetic information processing transcription, glycan biosynthesis and metabolism, and lipid metabolism	-	-
Zuo et al. (28)	**-**	Hexacosanedioic acid, Cyclophosphamide (18:1(11Z)/0:0), Lysophosphatidylethanolamine (0:0/18:2(9Z,12Z), 20:0/0:0, etc.), lysophosphatidylcholines (18:0, 15:0, etc.), Palmitoyl-L-carnitine, N-stearoyl glutamic acid, Phosphocholine, Oleamide, Linoleic acid were increased in hypertension; 6-Hydroxynicotinic acid, L-Leucine, Guanidineacetic acid, Coprocholic acid, Riboflavin, 2-Oxo-4-methylthiobutanoic acid, Vitamin D3, Decenedioic acid, α-Tocotrienol, Pantothenic Acid, Lysophosphatidic acid (0:0/18:0), tetracosahexaenoic acid, Vitamin D6, pipecolic acid, 3-Indoleacetic Acid, MG(0:0/24:6(6Z,9Z,12Z,15Z,18Z,21Z)/0:0, 0:0/22:0/0:0, etc.), Eicosanedioic acid, Vitamin D5, corticosterone, N-stearoyl tyrosine, 8(S)-HETE, Coenzyme Q4 were decreased in hypertension	-
Kim et al. (22)	**Up-regulated in hypertension:** LPS biosynthesis, steroid degradation, ABC transporters, PTS, and bacterial secretion system **Down-regulated in hypertension:** ubiquinone and other terpenoid-quinone biosynthesis, beta-alanine metabolism, selenocompound metabolism, cyanoamino acid metabolism, d-alanine metabolism, one carbon pool by folate, riboflavin metabolism, and folate biosynthesis	Plasma butyrate levels were lower in hypertension;Plasma levels of I-FABP, LPS, Th17 cells were higher in hypertension	-
Jin et al. (21)	**Up-regulated in hypertension:** DNA-binding protein, Dehydrogenase, regulatoR, membrane, oxidoreductase, Transposase, Transcriptional regulator, Mate efflux family protein, radical SAM domain protein, hydrolase family 3, ABC transporter, Methyltransferase **Down-regulated in hypertension:** Hydrolase family 2, hydrolase family 43, integrase, Releases the supercoiling and torsional tension of DNA, tonB-dependent Receptor, DNA binding domain, excisionase family, integrase family, TonB dependent receptor, Phage integrase family, peptidase_, Histidine kinase, acetyltransferase, Efflux transporter rnd family, mfp subunit, Ragb susd domain-containing protein, Glycosyl transferase, family 2, Membrane, Transporter, helicase, domain protein, n-acetylmuramoyl-l-alanine amidase, transposase, Hydrolase	-	-
Yan et al. (19)	**Up-regulated in hypertension:** Membrane transport, LPS biosynthesis, steroid degradation, and enzymes involved in TMA production **Down-regulated in hypertension:** metabolism of other amino acid, cofactors and vitamins (including folate biosynthesis and metabolism, riboflavin metabolism, and ubiquinone biosynthesis), and SCFA-producing enzymes	-	The number of hypertension-associated species showed stronger correlation to the severity of hypertension
**Hypertension and borderline hypertension/Pre-hypertension**			
Huart et al. (24)	-	Fecal SCFAs (acetate, butyrate, and propionate) levels were higher in hypertension and borderline hypertension.	-
Han et al. (20)	-	-	Increasingly pervasive virus-bacteria linkages were found from healthy people to pre-hypertension people to hypertension patients
Li et al. (18)	**Up-regulated in hypertension:** LPS biosynthesis and export, phospholipid transport, PTS, biosynthesis of phenylalanine and phosphatidylethanolamine, and secretion system **Down-regulated in hypertension:** branched-chain amino acid biosynthesis and transport, ketone body biosynthesis, two-component regulatory system, and degradation of methionine and purine	Serum levels of phosphatidylserine, 3,4,5-trimethoxycinnamic acid, lysophosphatidylcholine, S-carboxymethyl-L-cysteine, and lysophosphatidylethanolamine were lower in hypertension and pre-hypertension	-
**Preeclampsia**			
Chang et al. (30)	**Up-regulated in preeclampsia:** LPS biosynthesis **Down-regulated in preeclampsia:** GPCR pathway	Fecal levels of butyric and valeric acids were lower in preeclampsia	-
**Pulmonary arterial hypertension**			
Kim et al. (31)	**Up-regulated in PAH:** Pathways for the synthesis of several amino acids, including arginine, proline, lysine, homoserine, methionine, ornithine, and tryptophan **Down-regulated in PAH**: anaerobic energy metabolism, gluconeogenesis, isoprene bio-synthesis, etc.	-	-

*GPCR, G protein-coupled receptor; I-FABP, intestinal fatty acid binding protein; LPS, lipopolysaccharide; PAH, pulmonary arterial hypertension; PTS, phosphotransferase system; SBP, systolic blood pressure; SCFA, short chain fatty acid; Th17, T helper 17; TMA, trimethylamine*.

### Alterations of Nutritional and Immunological Factors in Hypertension

Five studies (22, 24, 28–30) reported fecal metabolites alterations in hypertension (Table 4). Most studies focused on SCFAs, including acetate, butyrate, and propionate, etc., and related findings were summarized accordingly. Hypertension showed increased fecal SCFAs levels (24, 29) and decreased plasma SCFAs levels (22, 29). However, as opposed to these results, a preeclampsia study suggested that fecal levels of butyric and valeric acids were lower in preeclampsia (30). One metabolomics research reported multiple metabolites were altered in hypertension, such as lipids, acids, vitamins, etc. (28). Additionally, three studies reported immunological factors alteration in hypertension: pro-inflammatory cytokines, including TNF, IL-6, etc., and compounds, e.g., LPS, were increased (22, 33); anti-inflammatory compounds, such as 3,4,5-trimethoxycinnamic acid, were decreased in hypertension (18).

### Alterations of Gut Microbial Interactions in Hypertension

Two studies (19, 20) did microbial interaction analysis of gut microbiota (Table 4). The virome study reported co-occurrence networks linking viruses and bacteria and found increasingly pervasive virus-bacteria linkages from healthy individuals to pre-hypertension individuals and to hypertension patients (20). In another research, the number of hypertension-associated species showed stronger correlation to the severity of disease (19).

## Discussion

In the present study, we systematically reviewed human studies evaluating alterations of gut microbiota in hypertension and summarized major findings of gut microbiota dysbiosis including diversity, major taxa with disparate representation, microbial function, nutritional and immunological factors, and interactions.

Reduced microbial diversity is commonly recognized as a marker of microbial dysbiosis and many diseases show a dysbiosis characterized by a lower diversity (7). Our study found decreased diversity of gut microbiota in hypertension (18, 19, 33), pre-hypertension (18), preeclampsia (30), and PAH (31), although a contrary conclusion was also seen (26). In line with human study results, reduced microbial diversity also occurs in animal models of hypertension, such as spontaneously hypertensive rat (SHR) (35) and hypertensive obstructive sleep apnea (OSA) rats (36). Additionally, microbial community structure was altered in hypertension as reported in 11 enrolled studies. These findings have emphasized the gut dysbiosis phenomena in hypertension.

Another important assessment for microbiota dysbiosis was to explore differential taxa by comparing hypertension group and healthy control group. According to the available studies, SCFAs producers were depleted and some *Proteobacteria* and *Bacteroidetes* members were enriched in hypertension. Similarly, decrease of bacteria that produced SCFAs was seen in SHR (35) and hypertensive OSA rats (36). Furthermore, animal studies showed that acetate (37) and propionate (38, 39), two common SCFAs, were associated with reduced blood pressure and showed cardiovascular protective effects. These findings indicated that SCFAs-producing bacteria may be involved in the pathogenesis of hypertension.

Nutritional and immunological factors, which may reflect host-microbiota crosstalk, were summarized and we found that fecal SCFAs levels were increased (24, 29) and plasma SCFAs levels were decreased (22, 29) in hypertension. SCFAs, including acetate, propionate, and butyrate, are produced by bacterial fermentation of unabsorbed carbohydrates in the colon (7, 40). Then SCFAs are secreted into the gut lumen, transported across the epithelial barrier, and transported to the bloodstream (41). As mentioned above in the present study, depletion of SCFAs producing bacteria and increased fecal SCFAs levels in hypertension indicated less SCFAs production and more excretion; meanwhile, less SCFAs transportation to the bloodstream and lower level of circulating SCFAs was observed (22, 24, 29). An animal study showed reduced expression of butyrate transporter in the colon in SHR and this may explain the elevated fecal SCFAs levels and diminished SCFAs levels in circulation (42). Furthermore, blood pressure reduction was observed with the chronic intervention of acetate in mineralocorticoid excess–treated mice (37). In terms of microbe-host interactions, SCFAs can influence host cells by interacting with host G protein-coupled receptors (GPCRs), including Gpr41 and Olfr78, etc. to modulate blood pressure (43). Animal studies showed that reduced intestinal absorption and delivery of SCFAs were seen in GPR41-deficient mice (44) and hypotensive response of SCFA in wild-type mice could be modulated by disruption of Olfr78 and Gpr41 expression (38). Hence, SCFAs can modulate blood pressure via GPCRs. Accordingly, GPCR pathways were down-regulated as reported in one preeclampsia study (30). These findings support the role of SCFAs in blood pressure regulation and demonstrate that poor absorption and high excretion of SCFAs may play an important role in the pathogenesis of hypertension. This will provide new insights into SCFA-targeted therapies to manage blood pressure.

Another important finding concerning inflammation is that LPS levels increased in hypertension. LPS is an abundant component within the cell wall of Gram-negative bacteria and can stimulate the release of inflammatory cytokines, inducing inflammatory response (45). A critical role for inflammation in regulation of blood pressure was seen in prospective human studies and interventional animal studies (46–49). Consistent with this hypothesis, we found that, based on the function prediction analyses, up-regulation of LPS biosynthesis was observed in hypertension group (18, 19, 22); increase of LPS in plasma of hypertension cases was reported, indicating increased intestinal inflammatory response in hypertension (22).

Among the enrolled studies, heterogeneity of results was observed. The heterogeneity of these findings may be due to selection difference of hypertension cases and controls. As mentioned above, according to the risk of bias assessment using NOS, potential lack of representativeness of participants and selection bias were seen. Furthermore, gut microbiota composition could be influenced by various factors, of which the common ones are diet and medication factors. Thus, subjects in hypertension group and control group may have imbalanced characteristics of these factors, leading to heterogeneity of research results.

## Strengths and Limitations

To the best of our knowledge, the present study is the first systematic review to evaluate gut microbiota dysbiosis in human hypertension. We reported alterations of microbial diversity, major taxa with disparate representation, microbial function, nutritional and immunological factors, and interactions based on the enrolled studies with 9,085 participants.

We acknowledge some limitations. First, according to the risk of bias assessment, potential lack of representativeness of participants and selection bias were seen. Thus, summarized results should be interpreted with caution. Second, the diagnostic criteria for hypertension in different enrolled studies were different and this may contribute to inconsistent results of microbiota evaluation. Third, the articles which were not published in English or Chinese and did not provide English or Chinese abstracts were excluded in the present study; although only 11 studies were excluded in our study due to this reason, the generalizability of our findings might be limited. Additionally, most of the enrolled studies were conducted in China and the US and the extrapolation to other populations requires cautious interpretation.

## Conclusion

In conclusion, gut microbiota dysbiosis was observed in hypertension, including decreased diversity, altered microbial structure, compositional change of taxa, alterations of microbial function, nutritional and immunological factors, and interactions. Poor absorption and high excretion of SCFAs may play an important role in the pathogenesis of hypertension. Future studies on microbial-based therapies of hypertension are needed.

## Data Availability Statement

The raw data supporting the conclusions of this article will be made available by the authors, without undue reservation.

## Author Contributions

YG and BY structured and designed the study. YG and XL performed the literature search, data extraction, and wrote the first draft of the manuscript. YG and ZW evaluated the risk of bias. BY and ZW critically reviewed the manuscript. All authors approved the final version.

## Conflict of Interest

The authors declare that the research was conducted in the absence of any commercial or financial relationships that could be construed as a potential conflict of interest.
