# Supplementary material for: Gut Microbiota Dysbiosis in Human Hypertension: A Systematic Review of Observational Studies
Source: Front Cardiovasc Med. 2021 May 14;8:650227. doi: 10.3389/fcvm.2021.650227 (PMC8160125; doi:10.3389/fcvm.2021.650227)
Supplement: Supplementary file 1 [file Table_1.docx]

Supplementary Material

## Supplementary Table 1. Literature search strategy

| **Database** | **Search strategy** | **Date of search** | **Number of results** |
| --- | --- | --- | --- |
| PubMed  < Database inception – present > | ((Hypertension [Mesh]) OR (high blood pressure [Title/Abstract]) OR (hypertensive [Title/Abstract]) ) AND (Microbiota [Mesh] OR "microbiome"[Title/Abstract] OR "microbiota"[Title/Abstract] OR "microﬂora"[Title/Abstract] OR "bacterial ﬂora"[Title/Abstract] OR "bacterial community"[Title/Abstract] ) | March 26, 2021 | 361 |
| EMBASE  < Database inception – present > | 'hypertension'/exp AND ('microbiome':ti,ab,kw OR 'microbiota':ti,ab,kw OR 'microﬂora':ti,ab,kw OR 'bacterial ﬂora':ti,ab,kw OR 'bacterial community':ti,ab,kw) | March 26, 2021 | 2267 |
| Web of Science  (SCI-EXPANDED, SSCI, A&HCI, CPCI-S, CPCI-SSH, ESCI, CCR-EXPANDED, IC)  < Database inception – present > | (TS=(hypertension) OR TS=(“high blood pressure”) OR TS=(hypertensive)) AND (TS=(microbiome) OR TS=(microbiota) OR TS=(microﬂora) OR TS=(“bacterial ﬂora”) OR TS=(“bacterial community”) ) | March 26, 2021 | 1055 |
